# Portuguese adaptation of the Chronic Heart Failure Knowledge Questionnaire (KQCHF)

**DOI:** 10.1186/s12872-023-03325-5

**Published:** 2023-06-19

**Authors:** Ana Paula Azzam, Tatiane Fidelis, Andreia Nunes, Rui Valdiviesso, Teresa Limpo, Emília Moreira, José Silva-Cardoso, São Luís Castro

**Affiliations:** 1 Faculty of Psychology and Educational Sciences of the University of Porto, Rua Alfredo Allen, S/N, 4200-135 Porto, Portugal; 2 Faculty of Nutrition and Food Sciences of the University of Porto, Porto, Portugal; 3 CINTESIS@RISE, MEDCIDS, Faculty of Medicine of the University of Porto, Porto, Portugal; 4 CINTESIS@RISE, Knowledge Management Unit, Faculty of Medicine of the University of Porto, Porto, Portugal; 5 CINTESIS@RISE, Department of Medicine, Faculty of Medicine of the University of Porto, Porto, Portugal; 6 Department of Cardiology, University Hospital Center of São João, Porto, Portugal

**Keywords:** Heart failure, Knowledge of the disease, Health literacy, Instrument validation

## Abstract

**Background:**

A patient’s knowledge of heart failure (HF) is associated with better outcomes. The more information patients have about their illness, the less likely they are to be readmitted to the hospital. Such knowledge includes the cause, symptoms, probable duration, and expected evolution of the clinical picture. In Portugal, a tool for testing patient knowledge is an unmet need. Therefore, this study aimed to adapt and test the Chronic Heart Failure Knowledge Questionnaire (KQCHF) for the Portuguese context.

**Methods:**

This work includes three cross-sectional studies. In Study 1, subjects were divided between before and after receiving information about HF. In Study 2, participants answered the questionnaire before and after reading the brochure. In Study 3, KQCHF was applied to patients with HF. Studies 1 and 2 were carried out in the general population. Study 3 was carried out with HF outpatients. Convenience sampling was applied to participants in the three studies.

**Results:**

In Study 1 (*n* = 45), those who received information had better scores (9.2 ± 1.9) than those who did not (6.0 ± 2.3). In Study 2 (*n* = 21), the scores were higher after reading the brochure (10.4 ± 1.7 vs. 6.5 ± 2.9). In Study 3 (*n* = 169), women had better scores than men (9.1 ± 2.1 vs. 8.3 ± 2.2, overall: 8.5 ± 2.2), and knowledge was correlated with education (*r* = .340, *p* < .001) and age (*r* = -.170, *p* = .030).

**Conclusion:**

The Portuguese adaptation of KQCHF captured relevant knowledge about HF and has shown promising results for clinical and research purposes. The questionnaire can be useful in assessing HF patients’ knowledge of their disease and as a basis for the implementation of general and personalised educational strategies to improve HF knowledge and, therefore, promote health literacy and self-care.

**Supplementary Information:**

The online version contains supplementary material available at 10.1186/s12872-023-03325-5.

## Background

Heart failure (HF) is a condition in which the heart cannot maintain an adequate blood supply to meet the needs of the tissues [1, 2]. This may occur during the final stage of many forms of chronic cardiopathies. To regulate cardiac output, compensation mechanisms such as cardiac or neurohumoral hypertrophy, sympathetic autonomic nervous system, and atrial natriuretic peptide can be activated [3, 4]. However, the ability to compensate for these mechanisms is limited. Thus, the origin of HF is characterized by a decrease in cardiac output and tissue perfusion or an accumulation of blood in the veins, causing peripheral and pulmonary oedema [5].

Studies on cardiovascular diseases have shown that HF is the main cause of hospitalisations in people over 65 years of age in Europe [6, 7]. In both Europe and the United States, between 5.0% and 9.0% of people over 65 years of age suffer from HF. In Portugal, 4.4% of all people between 25 and 99 years of age have this condition. In the population between 70 and 79 years, the percentage increases to 12.7%, and among those over 80 years old, the numbers reach the mark of 16.1% [8].

A crucial factor in the treatment of HF is the information that patients have about the disease. Through this knowledge, patients can foresee some signs and symptoms of the disease and know the directions to follow to maintain a healthy lifestyle. Individuals who have difficulty maintaining self-care are more likely to be hospitalized and consequently have a low life expectancy [5, 9]. Therefore, it is relevant to have measures to assess the knowledge of patients about HF. Therefore, this study aims to develop and validate a questionnaire to assess the knowledge of individuals about HF in the Portuguese context.

### The importance of knowledge

Adherence to treatment is characterised as a behaviour in which the person can integrate medical orientation appropriately into their daily life (e.g., use of medications, diet, lifestyle changes). Although there is little research on the importance of knowledge of HF in other chronic diseases, studies have consistently demonstrated the positive impact of patient knowledge on achieving a favorable prognosis [10, 11]. For example, diabetes is a disease in which the individual needs specific knowledge to improve adherence to treatment by introducing appropriate lifestyle changes (e.g., diet, physical exercise) to control glycaemic levels [12]. By acquiring this knowledge, diabetes patients can mitigate the risk of acute complications (e.g., limb amputation), thus reducing the risk of mortality [13]. However, although these factors are characterised as protective health behaviours, research shows that low adherence to treatment in chronic diseases is a worldwide problem. In developed countries, it can reach up to 50.0%, and in other countries still in development, it can be even more concerning [14].

Regarding self-care in diabetes, the American Diabetes Association designates two national standards of education and support for self-management of the disease. The first is diabetes self-management education (DSME), which is characterized by a continuous process of learning and developing skills related to self-management of the disease [11]. The main objective of the DSME is to support the patient in conscious decision making, that is, to provide the necessary information about the disease, to improve their health status and quality of life. The second national standard is Diabetes Self-Management Support, which includes a variety of behavioural, educational, psychosocial, or clinical activities that help patients with diabetes implement the strategies needed to manage the new condition continuously and effectively [4, 15, 16]. According to healthcare guidelines [17], DSME is essential so that patients learn the necessary skills and can maintain information after receiving the diagnosis. In a cross-sectional study conducted with 120 patients with type 2 diabetes, it was concluded that factors such as self-care, nutrition, self-management of glycaemic control, and guided self-medication were significantly associated with improved quality of life [18]. Therefore, it is evident that interventions implemented for health literacy can positively impact the patient’s prognosis [19].

Despite the limited availability of information highlighting the importance of knowledge about HF in comparison with other diseases, studies suggest that it may also be relevant to evaluate the knowledge of patients about their clinical status [14–16]. Given the challenging nature of diagnosing HF, which often occurs in advanced stages rather than in the early stages, it is crucial that patients receive accurate and vital treatment information once diagnosed. The more information patients have about their disease, the lower the chances of readmission to the hospital. Health literacy (or health knowledge) includes the cause, symptoms, probable duration, and expected evolution of the clinical condition [7, 11].

### Evaluation of knowledge about HF

Given the limited availability of materials produced and validated in the Portuguese language to evaluate knowledge in HF, a review of medical literature was conducted to identify the most suitable instrument for this study. In this review, five questionnaires were discarded (cf. Table 1) for the following reasons: *(1) language difficulty (specific medical terms), (2) gap-filling questionnaires which would require more time and specific knowledge by the participants, (3) poor organization, and (4) comprehensiveness of the information about the disease.*

**Table 1:** Review of the instruments used to evaluate knowledge related to heart failure

Authors	Instrument title	Characteristics
Dewalt et al. (2004) [9]	Knowledge of the CHF questionnaire	A 15-question scale was administered verbally to each patient. Multiple choice questions and true/false responses
Ghisi et al. (2015) [20]	The second version of the Coronary Artery Disease Education Questionnaire—CADE-Q II	31 elements, each with 4 multiple choice options referring to medical conditions (7 items), risk factors (7), physical exercise (7), nutrition (7), and psychosocial risk; (7)
Artinian et al. (2002) [21]	Questionnaire about Heart Failure Patients’ Knowledge of Their Disease	13 multiple choice questions, plus two gaps filling items related to diet (3), medication (5), self-care (3), concepts related to HF (2) and signals and symptoms (2)
Van der Wal et al. (2005) [22]	Dutch Heart Failure Knowledge Scale	15 multiple choice questions related to HF in general (4), HF treatment (6 items related to diet, fluid restriction, and physical activity), symptoms, and recognition of symptoms (5)
Kato et al. (2013) [23]	Japanese Heart Failure Knowledge Scale	17 elements referred to HF in general (3), signals and symptoms of HF (5), and treatment and self-care related to HF (9)
Hui Yang et al. (2006) [24]	Congestive Heart Failure Knowledge Test and Social Support Research Questionnaire	**[1]. Congestive HF Knowledge Test** (10 multiple choice items), Chronic heart failure questionnaire (20 items divided into 4 domains: dyspnoea (5), fatigue (4), emotional state (7) and domain (4), with each item scored on a 7-point Likert scale (1 = worse/ 7 = better). **[2]. Social support questionnaire** (an independently completed questionnaire of 20 items that measures self-perception of social support adequacy in patients with chronic disease patients.)
Bonin et al. (2014) [25]	Patients with Heart Failure Questionnaire	19 elements divided into areas of importance for patient education: physical exercise (4), medication (2), concept of signals and symptoms (1), lifestyle and risk factors (2), diagnosis (1), treatment (1), self-care and lifestyle (2), signals, symptoms and self-care (1), pathophysiology (1), self-care (2), signals, symptoms and risk factors (1), risk factors (1)

It was verified that the most appropriate instrument for this study was the Chronic Heart Failure Knowledge Questionnaire (KQCHF), built by DeWalt et al. [9], as it was designed for patients with low levels of education and could thus be adapted to the diverse population of this study. This questionnaire assesses HF knowledge and treatment using 14 multiple choice questions ranging between 3 and 5 possible answer choices, including the “I don’t know” option to avoid random answers.

Since it is understood that perception of the disease leads the patient to handle their treatment in a more positive way, the KQCHF addresses the themes of dehydration and signs that indicate a worsening of the clinical condition. In the last part, the patient should know the consequences and possible preventive measures to be taken in everyday situations, such as if the legs are more swollen than normal, shortness of breath, or weight gain.

DeWalt et al. [9] validated this questionnaire in a study implemented to promote self-care in HF in a population of patients with low levels of education. This was achieved using randomized clinical trials that demonstrated that educational programs could reduce hospitalisations and improve prognosis in patients with HF. In the pilot test, focus groups and individual cognitive response interviews were conducted to develop an educational manual for these patients. After this process, an intervention was carried out for the management of the disease, including the use of an informative brochure, an individualised one-hour educational session, and scheduled phone calls, which were reduced in frequency over six weeks. After the pilot test, a 3-month study was conducted to test the efficacy and acceptability of the program.

The results showed that the percentage of patients who weighed themselves every day increased from 32 to 100% in 12 weeks. Furthermore, the increase in the Minnesota Living with Heart Failure Scale (MLHFS) was 9.90 points for three months (95%, CI = 0.5, 19.2). Improvements in New York Heart Association HF functional class were also observed. At the end of the study, it was concluded that the HF management program specifically designed for patients with low levels of education is acceptable and is associated with improved self-care behaviour and disease-related symptoms.

### The importance of the study

Poor levels of knowledge about diseases can cause higher rates of rehospitalisation and even death. HF is one of the leading causes of death in Portugal [26] and cardiovascular disease is the leading cause of death worldwide [27]. Therefore, the validation of this questionnaire represents great use for health professionals and patients. This research was conducted to provide support and create a new tool for healthcare professionals to better understand and support patients who were suddenly diagnosed with an unexpected pathology.

## Aims and objectives

**Fig. 1:**
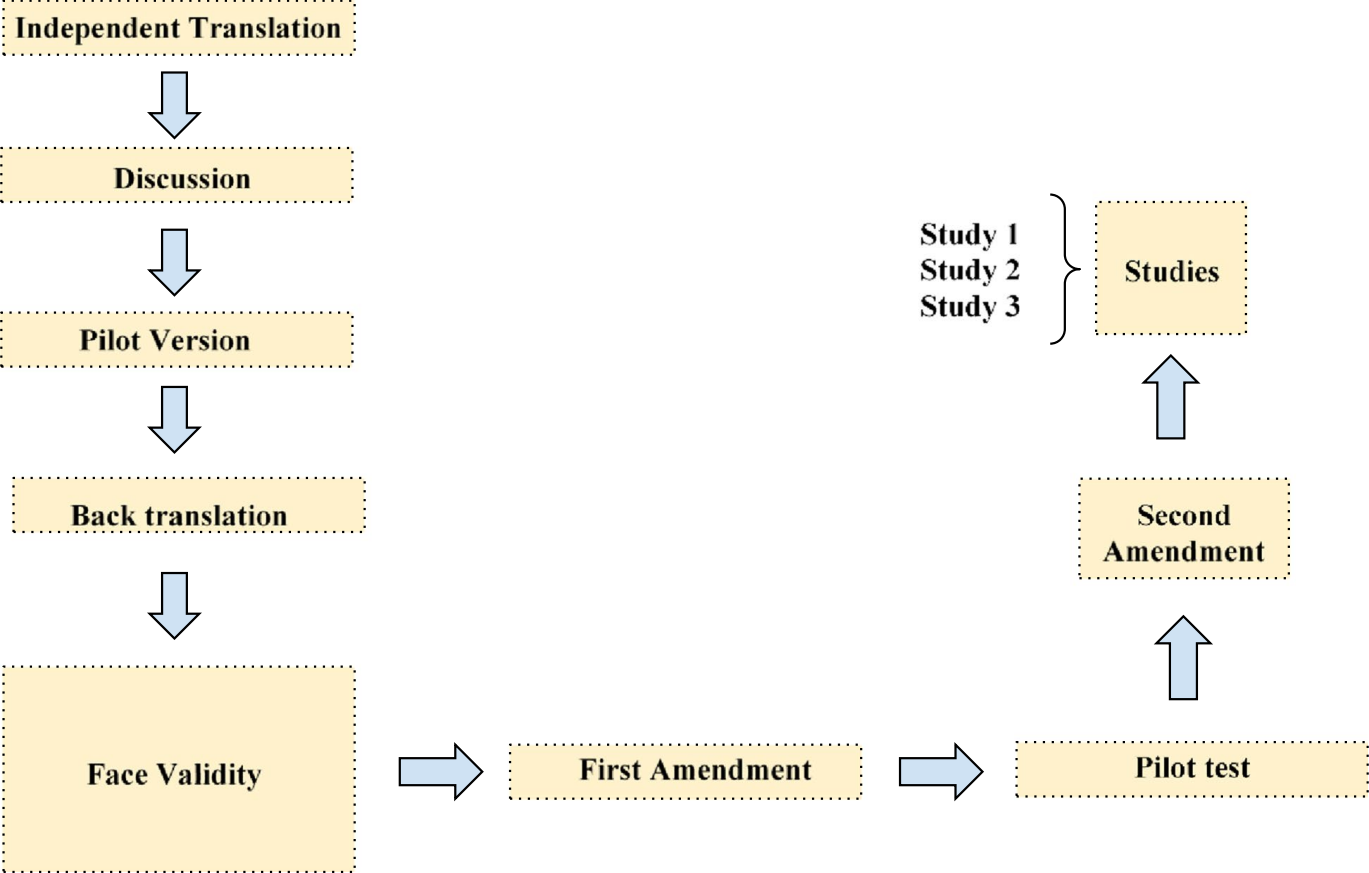
Development stages of the instrument translation and adaptation to Portuguese

This research aimed to develop and test the Portuguese version of the KQCHF. To do this, we carried out six stages of instrument adaptation, followed by three studies (cf. Fig. 1).

## Methods

### Adaptation of the instrument

The questionnaire was initially independently translated by two researchers from the Centre for Psychology of the University of Porto (CPUP). At the end of the translation process, two versions were obtained. Both versions were analysed and compared to match the final version (cf. Supplemental Material 1). To ensure the validity of the translation of the questionnaire, a back-translation of this version was performed by a Native American and European Portuguese speaker. This version confirmed the semantic and content similarity of the Portuguese version to the original English version (cf. Supplemental material 2).

To ensure the face validity of the instrument, a group of five cardiologists analysed and evaluated the first version of the questionnaire. Therefore, they were asked to examine all items and answer three questions using a 5-point Likert scale (*1* = *totally disagree; 5* = *totally agree*): (1) This questionnaire assesses what people know about heart failure; (2) This questionnaire consists of items that address important aspects of heart failure; (3) This questionnaire seems to be a good indicator of people’s knowledge about heart failure.

Cardiologists suggested two amendments, both of which were implemented. In the first amendment, they suggested removing item 14 from the original scale (“Compared to someone without heart failure, a person with heart failure should drink…”) as its content concerns a specific phase of HF, which affects only a portion of patients. The second change was related to item 10 (“Is weight gain a sign that heart failure is getting worse?”), which was considered vague. This was reformulated to make it more specific, becoming “Is unexpected, progressive, and rapid weight gain a sign that heart failure is getting worse?”.

Regarding the evaluation of the instrument by cardiologists, all questions were scored with the highest score (“I totally agree”), except for the third question *(“This questionnaire seems a good indicator of people’s knowledge of heart failure”)*, which was evaluated by one of the cardiologists with the value four (“I agree”).

To test the applicability of the questionnaire, a pilot test was performed consisting of a group of patients with HF in a hospital setting. During this test, it was found that item 1 *(“Heart failure means…”)* caused too much anxiety in participants due to the second response option *(“your heart may stop beating at any time”).* For this reason, it has been decided to change the order of the responses, moving this option from number two to number three. Furthermore, an adjustment was made to the position of item 3 in the general context of the questionnaire *[“Medication prescribed to urinate may cause the patient to become dehydrated (losing too much water). Which of the following signs indicates dehydration?”]*, which passed to the end of the questionnaire, to facilitate the coherence and integration of the items (c.f. The final version of the instrument is presented in Supplementary Material 1).

In addition to the KQCHF, an HF information brochure was also used, which was developed by the multidisciplinary team of the Deus ex Machina (DeM) Project. The brochure contained important information on HF, including the information described in the questionnaire, such as the definition of the disease, warning signs, and how to proceed after diagnosis (cf. Supplemental Material 2).

### Studies

#### Study 1

This study was carried out on April 2017 and aimed to evaluate the ability of KQCHF to differentiate between those with different levels of knowledge. The hypothesis of this study was that the questionnaire would be able to capture interindividual differences regarding the knowledge of heart failure.

##### Participants

Participants were selected by convenience sampling between attendants at a large public display of the University of Porto activities aimed at general audiences. Exclusion criteria were being younger than 18 years and having HF, as it was assumed that HF patients would already have received some prior information on the disease.

##### Experimental design and procedure

In this experimental procedure, the aforementioned informative brochure was used, in order to test whether the responses to the questionnaire would improve after the participants received more knowledge about HF.

Participants were randomly divided into two experimental conditions regarding the order the instruments were introduced: brochure-questionnaire (B-Q) vs. questionnaire-brochure (Q-B). In the B-Q group, a brief presentation on the study was made and the informative brochure with basic data on HF was presented. Subsequently, participants were asked to complete the questionnaire independently. In the Q-B condition, the reverse procedure was followed: individuals were initially asked to complete the HF questionnaire independently and subsequently were presented with the brochure. With this methodology, participants who did not receive previous information of HF could be differentiated from those who did. Experimental conditions were compared regarding the frequency of correct answers using Pearson’s Chi-square test or Fisher’s exact test where applicable. A t-test for independent samples was performed to compare the average correct responses between the groups.

#### Study 2

This study aimed to evaluate if the questionnaire would be able to capture intraindividual differences regarding the knowledge of heart failure. To this end, an intrasubject experimental study, which was carried out between June and July 2017, was conducted in which participants had a longer time than in Study 1 to read the information contained in the brochure and then fill out the questionnaire independently again. Through this measure, the hypothesis was that the questionnaire would be able to capture increments in knowledge at the second moment, after participants were better informed about HF.

##### Participants

The participants were randomly selected between students from the Neurocognition and Science Lab at the University of Porto. As in the previous study, the presence of HF or a family member with HF was defined as an exclusion criterion.

##### Experimental design and procedure

In this study, an intrasubject experimental design was used, resorting to a t-test for paired samples in which the average of correct answers was compared before and after reading the brochure. All participants performed the same tasks. First, the individuals answered the questionnaire without receiving any information about the disease. The experimenter then gave the brochure with basic data on HF used in Study 1 to the participants, giving them 5 min to read it. Finally, after collecting the brochure, they were asked to respond independently to the questionnaire.

#### Study 3

The study was carried out at the São João University Hospital Centre (UHCSJ), between September 2017 and July 2018, with patients waiting to attend scheduled external consultations. This study was conducted as part of the DeM project "Symbiotic technology for societal efficiency gains: Deus ex Machina", with the approval of the Hospital Ethics Committee, and its main objectives were to evaluate the knowledge of patients with HF about the disease, as well as to examine its relationship with their gender, age, and education.

##### Participants

We estimated the number of potentially eligible patients at 537, according to a study developed in a similar period on the same setting [28]. The following inclusion criteria were defined: being 18 years or older, HF validated diagnosis and follow-up in the Heart Failure and Transplant Services of the Cardiology Service of UHCSJ. The exclusion criteria were severe visual impairment and an inability to write and communicate clearly.

##### Materials

Information questionnaire and informative brochure used in previous studies.

##### Procedure

The questionnaire was administered in the waiting room of the HF outpatient clinic of the hospital centre. The researchers approached the patients, explained the research being carried out, and asked them if they would be interested in participating while waiting for the medical consultation. After their agreement, the questionnaire was answered and then the information contained in the brochure was given. Finally, if the participant showed greater interest in the investigation, the instruction given was to ask for more information from the nurse or doctor.

To evaluate the knowledge of patients about HF, descriptive statistics were used. Regarding the relationship with gender, a t-test was performed for independent samples. Depending on age and education, Pearson’s correlations were conducted to assess their relationship with the level of knowledge of participants with HF.

### Patients and public involvement

Patients and the public were not involved in conceptualising, planning, conducting, or analysing the research.

## Results

### Study 1

This study included 45 individuals (28 women), aged 18 to 66 years (41.6 ± 13.5). Of these, 2.2% had sixth grade, 4.4% had ninth grade, 20.0% had 12th grade, 40.0% had a college degree, and 33.3% had higher education.

Table 2 presents the frequency and percentage of correct responses for each item of the questionnaire according to the experimental condition. As seen in the table, in both conditions, item 13 had the lowest percentage of correct responses (0.0% in the Q-B condition and 4.5% in the B-Q condition). The items that had the highest percentage of correct responses were 2 and 11 in the Q-B condition (73.9%) and 1, 4 and 11, in the B-Q condition (95.5%). Correct answers for items 1, 4 and 10 were significantly more frequent in the B-Q condition.

**Table 2:** Frequency and percentage of correct answers in each item by experimental condition

Items	Correct answers in Questionnaire-Brochure condition		Correct Answers in the Brochure-Questionnaire condition		*p*-value
	Frequency	Percentage	Frequency	Percentage	
1	12	52.2%	21	95.5%	.002
2	17	73.9%	16	72.7%	.928
3	13	56.5%	14	63.6%	.626
4	15	65.2%	21	95.5%	.022
5	12	52.2%	17	77.3%	.079
6	5	21.7%	10	45.5%	.092
7	15	65.2%	18	81.8%	.314
8	7	30.4%	13	59.1%	.053
9	3	13.0%	3	13.6%	1.000
10	3	13.0%	19	86.4%	< .001
11	17	73.9%	21	95.5%	.096
12	14	60.9%	18	81.8%	.189
13	0	0.0%	1	4.5%	.489
14	1	4.3%	1	4.5%	1.000

*Q-B* Questionnaire before brochure, *B-Q* Brochure before questionnaire

To verify whether the results of the KQCHF were different between the two experimental conditions, a t-test was performed for independent samples in which the average correct responses were compared between the groups. The results showed that the participants in the Q-B condition (6.0 ± 2.3) answered correctly to fewer questions than the participants in the B-Q condition (9.2 ± 1.9), t (55) = -5.2*, p* < 0.001.

### Study 2

A total of 21 people (10 women) between the ages of 20 and 34 (25.9 ± 3.6) participated in this study, all with a university degree.

Table 3 presents the frequency and percentage of correct answers for each item of the questionnaire according to the experimental condition. In both conditions, item 13 had the lowest percentage of correct answers (8.6% in the Q-B condition and 26.0% in the B-Q condition). In the first moment, the item that obtained the highest percentage was 4 (86.9%). The B-Q condition demonstrated that the questionnaire was able to capture effective changes in HF knowledge, as five items represented the maximum percentage of correct answers.

**Table 3:** Frequency and percentage of correct answers in each item by experimental condition

Items	Correct answers in Q-B Condition		Correct Answers in the B-Q Condition	
	Frequency	Percentage	Frequency	Percentage
1	19	82.60%	22	100%
2	10	43.40%	14	60.80%
3	15	65.20%	18	78.20%
4	20	86.90%	22	100%
5	15	65.20%	22	100%
6	5	21.70%	14	60.80%
7	14	60.80%	18	78.20%
8	4	17.30%	14	60.80%
9	3	13.00%	5	21.27%
10	6	26.00%	22	100%
11	15	65.20%	21	95.60%
12	19	82.60%	15	65.20%
13	2	8.60%	6	26.00%
14	3	13.00%	22	100%

*Q-B* Questionnaire before brochure, *B-Q* Brochure before questionnaire

Intraindividual differences in answering the questionnaire were asserted by paired samples t-test, in which the average of correct answers was compared before and after reading the brochure. The results showed that, before reading the brochure, the participants answered fewer questions (6.5 ± 2.9) than after reading the brochure (10*.*4 ± 1.7), t (30) = *-*6*.*8, *p* < 0.001.

### Study 3

This study included 169 individuals (47 women), aged 32 to 87 years (62.6 ± 10.9), and a mean of 6.5 years of education (*SD* = 4.0). Tables 4 and 5 illustrate the items with higher frequency of correct answers and lower frequency of correct answers, respectively.

**Table 4:** List of questions with higher frequency of correct answers

Questions and Answer Options (correct answer in bold)	Frequency	Percentage
**1.** Heart Failure means that:		
Your heart is beating out of rhythm	49	29.00%
**Your heart is not pumping blood as it should**	**83**	**49.10%**
Your heart can stop beating at any time	14	8.30%
You’re having a heart attack	4	2.40%
I don’t know	19	11.20%
**2.** Which of the following symptoms may be due to heart failure?		
headaches	18	10.70%
yellow skin	6	3.60%
**shortness of breath then lying down**	**132**	**78.10%**
vomit blood	2	1.20%
I don’t know	11	6.50%
**3.** The medication prescribed for urination may lead to the patient becoming dehydrated (lost too much water). Which of the following signs indicates dehydration?		
**Dizziness**	**66**	**39.10%**
shortness of breath	6	3.60%
chest pain	9	5.30%
burning when urinating	46	27.20%
I don’t know	41	24.30%
**11.** If you eat too much salt, it will:		
**cause heart failure to worsen**	**166**	**98.20%**
have no effect on heart failure	3	1.80%
I don’t know	0	00.00%
**12.** What should you do when you feel more short of breath and your weight increases about 3 kg over your usual weight?		
stop taking diuretics	2	1.20%
**call your doctor**	**68**	**63.90%**
go on a diet	29	17.20%
weight yourself to see if you gained more weight	16	9.50%
I don’t know	14	8.30%

**Table 5:** List of questions with lower frequency of correct answers

Questions and Answer Options (correct answer in bold)	Frequency	Percentage
4. Is shortness of breath a sign that heart failure is getting worse?		
**Yes**	**19**	**11.20%**
No	143	84.60%
I don’t know	7	4.10%
**5.** Is swelling in the legs or ankles a sign that heart failure is getting worse?		
**Yes**	**18**	**10.70%**
No	135	79.90%
I don’t know	16	9.50%
**7.** Is waking up at night with shortness of breath a sign that heart failure is getting worse?		
**Yes**	**32**	**18.90%**
No	131	77.50%
I don’t know	6	3.60%
**8.** Is vomiting blood a sign that heart failure is getting worse?		
Yes	123	72.80%
**No**	**29**	**17.20%**
I don’t know	17	10.10%

The participants answered an average of 8.5 questions (*SD* = 2.2). More specifically, it was observed that most of the participants knew that the origin of HF lies in the loss of the heart’s ability to pump blood as it should (49.1%) and that one of its main symptoms is shortness of breath when lying down (78.1%). A total of 39.1% of the participants were able to identify symptoms related to dehydration. Furthermore, almost all patients have shown that salt intake has harmful effects on HF (98.2%) and more than half of them have the idea that weight gain and symptoms related to shortness of breath justify that the patient contacts his doctor (63.9%). However, few of them answered that shortness of breath is one of the symptoms that heart failure is getting worse (11.2%), and 84.6% of the sample incorrectly answered that it was not. Similarly, few participants indicated that waking up at night with shortness of breath is a sign that HF is getting worse (18.9%).

Although more than half of the participants responded that sudden weight gain (about 6.6 pounds higher than the usual weight) justifies calling the doctor, only about a third answered that weight gain is a sign that HF is getting worse (34.3%) and that someone with HF should monitor their weight every day (24.3%), most of whom answered that people should weigh only once a week (39.1%).

Many participants erroneously reported that the worsening of the disease was associated with yellowish skin (57.4%), a symptom originally associated with jaundice (hepatitis); headaches (55.6%), which may be related to common sense that headaches would be the genesis of various related diseases; and vomiting blood (72*.*8%), a symptom that is also related to liver failure. Regarding Question number 3, the sample has also shown that it has little knowledge of the symptoms related to fluid retention (“swelling in the legs or ankles is a sign that HF is getting worse”—10.7% of correct responses), and, relatedly, about what to do when the legs swell more than normal (20.1% correct responses).

The responses to the questionnaire were significantly different between men and women, t(167) = 2.246, *p* = *0.026,* d = 0.38, with higher means for women (*M* = 9.1, *SD* = 2.1 vs *M* = 8.3, *SD* = 2.2). The results showed that education and knowledge were positively associated (*r* = *0.3*40, *p* < *0.0*01). Regarding age and knowledge, there was a weak negative correlation (*r* = -0.170, *p* = 0.030).

## Discussion

The main objective of the present investigation was to develop and validate KQCHF [9] for the Portuguese context. As presented in the Results section, there were a striking number of misconceptions of HF patients about the disease. In addition, providing information on the disease using brochures resulted in a higher level of knowledge about HF, classified according to our version of the KQCHF questionnaire.

Regarding studies 1 and 2, it is worth mentioning that the authors assumed that providing information through a brochure would improve knowledge and that these changes would be reflected in the questionnaire scores. Therefore, these studies were designed to test the ability of the questionnaire to detect knowledge changes, rather than to test the assumption that the brochure would improve the knowledge of participants on HF.

The present research found that education and HF knowledge were positively correlated. This was also found in other studies that established that higher education and older age were associated with higher levels of knowledge of HF [21]. As expected, the questionnaire was able to enhance the differences between participants who had received prior information on HF and those who had not received this information. This result demonstrates the relevance of promoting health literacy with patients, as well as improving patient engagement with their healthcare.

In study 3, patients were approached at the hospital to answer the Portuguese version of the KQCHF questionnaire. After reviewing the results, many misconceptions about the disease were found. This was observed considering the high frequency of the association of HF with yellow skin, headache, and vomiting blood, as well as the low frequency of correct responses regarding signs of oedema (i.e., swelling caused by fluids that accumulate in body tissues).

These misconceptions could lead to unwanted results, as a recent systematic review and meta-analysis evaluated the association between low literacy levels in HF and mortality in five different studies. The authors found a strong association between inadequate health literacy and mortality, with a 67% higher risk of mortality for those who had lower levels of health literacy. Inappropriate knowledge was also significantly associated with hospitalisation: By analysing eight studies, the authors found that low literacy levels increased the risk of hospitalisation by approximately 20% [29].

Regarding gender, the results showed that there were significant differences between men and women, and women had greater knowledge of HF. Other studies [30, 31] that mention the relationship between gender and knowledge showed nonsignificant results when comparing genders. For example, a study found that 69.3% of the sample had an adequate level of knowledge of HF, but without significant differences between men and women. Although the results are different, it is important to recognise that the studies had occurred in different countries and therefore the populations had different characteristics [32]. Therefore, data may vary according to the health care settings and information provided to patients by medical personnel.

It is evident that education, intervention, and support for self-management are key aspects of management and a good prognosis in HF. For patients, self-care it is essential during the path of disease, namely in worsening stages or when more advanced treatment is required [33]. In this process, the use of the questionnaire is essential as an assessment of the patient’s knowledge of their condition and is a great tool for healthcare providers to understand and help patients. If the questionnaire is used frequently and changes in patient habits are observed, it can be understood that the educational process of treatment can strengthen the feeling of improvement of the patient [26, 34]. It is also imperative that healthcare workers improve their education and receive specific training in the area to support the management of the disease [35].

However, it is important to consider that experiences in the period prior to the onset of the disease influence the beliefs of patients and their perception of treatment [6, 36]. Therefore, it is important to understand the characteristics related to the health and pathology in question, considering the collective and individual experiences of the patients [11, 14, 37].

In addition, it is also the responsibility of the medical team to assess the socioeconomic situation and the life story of the patient. Medical personnel should recognise that patients are active agents of change, acknowledging them as the protagonists of their own improvement [5, 15, 38].

### Limitations and strengths

This study addresses the identified need to have a questionnaire that can inform physicians and other caregivers about the knowledge of patients with heart failure about the disease. As a result, patients can receive better information and ultimately improve their own self-care. One of the main strengths of this study is the methodological robustness of the translation and adaptation of the questionnaire, which improves its precision and avoids bias. Furthermore, the input from cardiologists contributed to a better adaptation to the reality of patients. Two pilot studies contributed to test the ability of the questionnaire to represent changes in knowledge about heart failure. As the questionnaire is specifically adapted for the Portuguese context, the generalisability of these results is naturally compromised. In addition, this study was developed in a single setting, which may further compromise external validity. Another identifiable limitation is the small sample size of heart failure patients. Larger multicentre samples would allow for a wider generalisability of the results. Nevertheless, the methodological approach to this adaptation and testing may be useful in other cultural and social realities.

## Conclusions

The application of a Portuguese version of the KQCHF questionnaire in a small sample of HF patients revealed a general lack of knowledge about the disease and a dependence on education and sex on the classification of the questionnaire’s answers. In convenience samples from the general population, the KQCHF score was significantly higher in those who received information on HF using a brochure. These results suggest that the Portuguese version of the KQCHF may be useful in assessing the knowledge of HF patients about their disease and serving as a basis for implementing general and personalised educational strategies aimed at improving knowledge of HF and thus promoting health literacy and self-care. By doing this, patients can achieve autonomy in their treatment and take care of their health, increasing the chances of a good prognosis and successful management of the disease.

## Supplementary Information

**Additional file 1.** Final version of the questionnaire.

**Additional file 2.** Back translation.

## Data Availability

The data supporting the findings of this study are available from the DeM and AdHeart projects, but restrictions apply to the availability of these data, which were used under license for the current study and are therefore not publicly available. However, the data are available from the corresponding author (APA) upon reasonable request and with the permission of the promoting institutions.

## Abbreviations

HF: Heart failure
KQCHF: Chronic Heart Failure Knowledge Questionnaire
Q-B: Questionnaire-Brochure
B-Q: Brochure-Questionnaire
UHCSJ: University Hospital Centre of São João
CPUP: Centre for Psychology at University of Porto
DSME: Education in self-management of diabetes
DSMS: Diabetes Self-Management Support
