# Supplementary material for: Portuguese adaptation of the Chronic Heart Failure Knowledge Questionnaire (KQCHF)
Source: BMC Cardiovasc Disord. 2023 Jun 19;23:307. doi: 10.1186/s12872-023-03325-5 (PMC10280838; doi:10.1186/s12872-023-03325-5)
Supplement: Supplementary file 1 — Additional file 1. Final version of the questionnaire. [file 12872_2023_3325_MOESM1_ESM.pdf]

I. Final Version of the Questionnaire

Conhecimento da Insuficiência Cardíaca Crónica

(DeWalt et al., 2004)

Gostaríamos de saber o que sabe acerca da insuficiência cardíaca e do seu tratamento. Nesta folha estão algumas perguntas sobre este tema. Pedimos que nos diga qual é a resposta que pensa estar correta para cada uma das perguntas. Se não souber a resposta, não se preocupe, diga apenas "não sei".

1. Insuficiência Cardíaca significa que:

- ☐ o seu coração está a bater fora do ritmo
- ☐ o seu coração pode parar de bater a qualquer momento
- ☐ o seu coração não está a bombear sangue como devia
- ☐ está a ter um ataque cardíaco
- ☐ não sei

2. Qual dos seguintes sintomas pode ser devido à insuficiência cardíaca?

- ☐ dores de cabeça
- ☐ pele "amarelada"
- ☐ falta de ar quando está deitado
- ☐ vomitar sangue
- ☐ não sei

3. A medicação receitada para urinar pode levar a que o doente fique desidratado (perdeu demasiada água). Qual dos seguintes sinais indica desidratação?

- ☐ tonturas
- ☐ falta de ar
- ☐ dores no peito
- ☐ ardência ao urinar
- ☐ não sei

De seguida estão indicados alguns problemas. Terá de dizer se o surgimento de cada um deles (ou o seu agravamento, no caso de existir anteriormente) é um sinal de que a insuficiência cardíaca está a piorar. Se não souber a resposta, diga apenas "não sei".

4. A falta de ar é um sinal de que a insuficiência cardíaca está a piorar?

☐ Sim ☐ Não ☐ Não sei

5. O inchaço das pernas ou dos tornozelos é um sinal de que a insuficiência cardíaca está a piorar?

☐ Sim ☐ Não ☐ Não sei

6. A pele amarelada é sinal de que a insuficiência cardíaca está a piorar?

☐ Sim ☐ Não ☐ Não sei

7. Acordar de noite com falta de ar é sinal de que a insuficiência cardíaca está a piorar?

Sim Não Não sei

8. Vomitar sangue é sinal de que a insuficiência cardíaca está a piorar?

Sim Não Não sei

9. Ter dores de cabeça é sinal de que a insuficiência cardíaca está a piorar?

Sim Não Não sei

10. O aumento de peso é um sinal de que a insuficiência cardíaca está a piorar?

Sim Não Não sei

11. Se comer demasiado sal, isso:

irá fazer com que a insuficiência cardíaca piore

irá fazer com que a insuficiência cardíaca melhore

não terá qualquer efeito na insuficiência cardíaca

não sei

12. O que deve fazer quando sente maior falta de ar e o seu peso aumentou cerca de 3 kg acima do seu peso habitual?

parar de tomar os diuréticos

telefonar ao médico

fazer dieta

- pesar-se no dia seguinte para ver se ganhou mais peso
- não sei

13. O que deve fazer quando as suas pernas incham mais do que o normal?

- tomar uma dose extra de diuréticos
- caminhar mais
- comer mais sal
- comer mais proteína
- não sei

14. Alguém com insuficiência cardíaca deve pesar-se:

- todos os dias
- uma vez por semana
- uma vez por mês
- apenas se se sentir mal
- não sei
