# Supplementary material for: Portuguese adaptation of the Chronic Heart Failure Knowledge Questionnaire (KQCHF)
Source: BMC Cardiovasc Disord. 2023 Jun 19;23:307. doi: 10.1186/s12872-023-03325-5 (PMC10280838; doi:10.1186/s12872-023-03325-5)
Supplement: Supplementary file 2 — Additional file 2. Back translation. [file 12872_2023_3325_MOESM2_ESM.pdf]

***II. Back Translation***

**Knowledge of Chronic Heart Failure**

(DeWalt et al., 2004)

We would like to know what you know about heart failure and its treatment. On this sheet are some questions on this topic. We ask you to tell us the answer that you think is correct for each of the questions. If you do not know the answer, do not worry, just say "I do not know".

1. Heart Failure means that:

- ☐ Your heart is beating out of rhythm
- ☐ Your heart can stop beating at any time
- ☐ Your heart is not pumping blood as it should
- ☐ You're having a heart attack
- ☐ I don't know

2. Which of the following symptoms may be due to heart failure?

- ☐ headaches
- ☐ yellow skin
- ☐ shortness of breath then lying down
- ☐ vomit blood
- ☐ I don't know

3. The medication prescribed for urination may lead to the patient becoming dehydrated (lost too much water). Which of the following signs indicates dehydration?

- ☐ dizziness
- ☐ shortness of breath
- ☐ chest pain
- ☐ burning when urinating
- ☐ I don’t know

Some problems are listed below. You will have to say if the appearance of each of them (or their aggravation, if it exists before) is a sign that heart failure is getting worse. If you do not know the answer, just say "I do not know".

4. Is shortness of breath a sign that heart failure is getting worse?

☐ Yes ☐ No ☐ I don’t know

5. Is swelling in the legs or ankles a sign that heart failure is getting worse?

☐ Yes ☐ No ☐ I don’t know

6. Is yellow skin a sign that heart failure is getting worse?

☐ Yes ☐ No ☐ I don’t know

7. Is waking up at night with shortness of breath a sign that heart failure is getting worse?

☐ Yes ☐ No ☐ I don’t know

8. Is vomiting blood a sign that heart failure is getting worse?

☐ Yes ☐ No ☐ I don’t know

9. Having headaches is a sign that heart failure is getting worse?

☐ Yes ☐ No ☐ I don’t know

10. Is weight gain a sign that heart failure is getting worse?

☐ Yes ☐ No ☐ I don’t know

11. If you eat too much salt, it will:

☐ cause heart failure to worsen

☐ cause heart failure to improve

☐ have no effect on heart failure.

☐ I don't know.

12. What should you do when you feel more short of breath and your weight increases about 3 kg over your usual weight?

☐ stop taking diuretics

☐ call your doctor

☐ go on a diet

☐ weight yourself to see if you gained more weight

☐ I don't know.

13. What should you do when your legs swell more than normal?

☐ take an extra dose of diuretics

- walk more
- eat more salt
- eat more protein
- I don't know.

14. Someone with heart failure should weigh themselves:

- every day
- once a week
- once a month
- when you don’t feel well
- I don't know.
